# All Forms of Life Cellular

**Published:** 1891-09

**Authors:** 


### ALL FORMS OF LIFE CELLULAR.

All life is cellular; this is true of the lowest plant and of the most highly developed animal. In the unicellular organism all the functions of life must be performed by the one cell; it must absorb, digest and excrete. It must fecundate and reproduce its species. As we ascend the scale of development we find a greater number of cells in the body. Not only do the cells multiply in number, but there is a division of labor among them, and the more marked this differentiation becomes, the higher stands the organism. In man some cells take upon themselves the duties of digestion, others that of elimination; some are concerned in locomotion, others in cerebration; others reason from the facts thus recognized. Communities of cells, engaged in the performance of a certain duty or duties, constitute an organ; and these, with their paths of inter-communication, form our bodies. Health is maintained only when each of these various communities of workers does its duty fully. If the pancreas fail to elaborate its proper secretion, the food does not undergo the normal digestive changes, and the liver, the heart, the lungs, the brain, and in short the whole mass becomes diseased or out of health.

